# Effectiveness of school-based preventive interventions on adolescent alcohol use: a meta-analysis of randomized controlled trials

**DOI:** 10.1186/1747-597X-9-48

**Published:** 2014-12-13

**Authors:** Henriette Kyrrestad Strøm, Frode Adolfsen, Sturla Fossum, Sabine Kaiser, Monica Martinussen

**Affiliations:** Regional Centre for Child and Youth Mental Health and Child Welfare, Faculty of Health Sciences, UiT The Arctic University of Norway, 9037 Tromsø, Norway

**Keywords:** Alcohol prevention, Alcohol drinking, Adolescents, Meta-analysis

## Abstract

**Background:**

Preventive interventions for adolescents are an important priority within school systems. Several interventions have been developed, but the effectiveness of such interventions varies considerably between studies. The purpose of this study was to assess the effectiveness of universal school-based prevention programs on alcohol use among adolescents by using meta-analytic techniques.

**Method:**

A systematic literature search in the databases, PubMed (Medline), PsycINFO (Ovid), EMBASE (Ovid) and WEB of Science (ISI) was conducted to search for empirical articles published in the period January 1990 to August 2014.

**Results:**

In total, 28 randomized controlled studies with 39,289 participants at baseline were included. Of these 28 articles, 12 studies (*N* = 16279) reported continuous outcomes (frequency of alcohol use and quantity of alcohol use), and 16 studies (*N* = 23010) reported categorical data (proportion of students who drank alcohol). The results of the random effects analyses showed that the overall effect size among studies reporting continuous outcomes was small and demonstrated a favorable effect from the preventive interventions (Hedges’  = 0.22, *p* < .01). The effect size among studies reporting categorical outcomes was not significant ( = 0.94, *p* = .25). The level of heterogeneity between studies was found to be significant in most analyses. Moderator analyses conducted to explore the heterogeneity showed neither significant difference between the different school levels (junior high schools and high schools), nor between the varied program intensities (low, medium and high intensity programs). The meta-regression analyses examining continuous moderators showed no significant effects for age or gender.

**Conclusions:**

The findings from this meta-analysis showed that, overall, the effects of school-based preventive alcohol interventions on adolescent alcohol use were small but positive among studies reporting the continuous measures, whereas no effect was found among studies reporting the categorical outcomes. Possible population health outcomes, with recommendations for policy and practice, are discussed further in this paper.

## Background

Early onset of alcohol use is associated with problematic substance abuse in later adolescence [1–4]. The study of Health Behaviour in School-aged Children (HBSC) show that on average 39% have their first alcoholic drink at age 13 or younger [5]. The prevalence rates and consequences of underage drinking warrant a comprehensive public health approach, grounded in evidence-based preventive interventions and policy-making [6]. The *European status report on alcohol and health* noted that 40% of the European countries did not have a written national alcohol policy in 2009, whereas in most Western countries drug prevention in schools has been a top priority [7]. Alcohol use among adolescents is a major public health concern and the political will to address this problem is considerable [8]. A range of preventive interventions to reduce or postpone alcohol debut among adolescents has been developed, and schools are important settings for such programs because large numbers of adolescents may be reached while costs are kept relatively low. Numerous estimates have been made of the social costs of early alcohol use, indicating that school-based drug and alcohol prevention programs should be a good investment [9]. The European Action plan states that those countries that are most active in implementing evidence-based alcohol policies and programs will profit from substantial gains in public health and well-being, productivity, and social development [10].

Universal school-based prevention is aimed at all students, regardless of their level of risk for alcohol use [11]. However, it is unclear whether or not the universal prevention programs are, in fact, effective. Several literature reviews [6, 12–16] and meta-analyses [17–21] have been conducted in this field. Some well-designed studies have suggested that school-based programs have the potential to reduce alcohol use among adolescents, but at the same time research has indicated that most drug prevention programs have no effect [8, 17]. Tobler and colleagues [17] conducted a meta-analysis of 207 universal school-based drug prevention programs, including studies with alcohol use as an outcome variable. They stated that program delivery matters more than program content and characterized successful programs as being interactive; i.e. programs that actively involve students while also including peer leaders [17]. This finding was also supported by a recent Cochrane review of 53 studies/programs, which concluded that the content of programs varied and suggested that program delivery may be more important for the effectiveness of the intervention than specific content [16].

It is argued that school-based interventions are most efficacious for preventing and reducing alcohol use among adolescents when delivered as primary prevention programs to youths who have not yet begun to experiment with alcohol [12, 22, 23]. Evidence suggests that prevention programs need to be initiated prior to seventh grade and that they need to address the associated risks of early drinking [24, 25]. The overall aim of school-based prevention is generally to delay the onset of drinking or to reduce alcohol consumption frequency. However, producing a meaningful effect on drinking behavior through school programs is a difficult task. Some research findings suggest that interventions aimed at preventing alcohol use are not likely to be effective [26–28], yet it is argued that large proportions of the resources in the prevention field are, in fact, dedicated to programs that have little potential to prevent and reduce alcohol abuse [29]. There are limited findings supporting the “universality” of intervention effects on alcohol outcomes [6]. For instance, a meta-analysis conducted by Rundall and Bruvold in 1988, evaluating the effect of school-based prevention programs, reported both a low short-term effect ( = 0.11) and a low long-term effect ( = 0.12) on alcohol use behavior. They also found that school-based alcohol use prevention programs had more instances of producing no effect or negative effects when compared to smoking prevention programs [18]. Similar findings were reported by Tobler and colleagues [17], where significant results were obtained only in one out of three cases, showing an overall small effect size ( = 0.14).

The objective of the present investigation was to perform an up to date meta-analysis of well-controlled experimental studies examining the overall effects of universal school-based preventive programs on alcohol consumption among adolescents under the age of 18 years. Randomized controlled trials (RCT) have been found to yield larger program effects than studies using quasi-experimental designs [20, 30]. The majority of the existing reviews have included also non-randomized studies, whereas this paper aims to include only randomized studies, because RCTs in general have stronger internal validity than quasi-experimental designs [31]. Different moderator analyses were conducted. First, we wanted to test if the effects of interventions vary between different school levels (elementary-, junior high- and high-school). Programs targeting adolescents in junior high schools are found to be marginally more effective than those targeting adolescents in elementary or high schools [21]. The majority of adolescents begin drinking alcohol prior to reaching adulthood; therefore, prevention programs need to target school-aged children and adolescents before they have established expectations and beliefs surrounding alcohol consumption [32].

Tobler and colleagues [17] found that programs with a duration of 11 to 30 hours were significantly more effective than those with a duration of 10 hours or less. However, a systematic review conducted by Cuijpers in 2002 stated that there is no definite evidence that intense programs are more effective than less intensive programs. Gottfredson and Wilson [21] showed in their research that program with brief duration are generally as effective as those with longer duration. Due to these inconsistent conclusions in relation to how the number of program sessions (intensity) may impact the effect, we also wanted to test the intensity of the program [8, 17, 21].

Finally, we wanted to explore whether the effects of preventive interventions vary with age and gender [33]. The prevalence of alcohol drinking increases significantly between the ages of 11 to 15 [5], and boys are generally found to drink more often and in greater quantities than girls. It is therefore likely that the effect of programs may differ between age groups and gender [16, 34–36].

## Methods

### Inclusion and exclusion criteria

Several inclusion and exclusion criteria were used to identify studies. Studies were included if they: (a) evaluated universal school-based prevention programs; (b) used randomized controlled trial (RCT) design with a control group; (c) assessed alcohol use outcomes; (d) provided sufficient information to calculate between-group effect size estimates; (e) included participants with a mean age of less than 18 years at pre-test; and (f) were published in English between January 1990 and August 2014.

Studies were excluded if the interventions: (a) were not described; (b) were designed for selective groups; or (c) were based on family and community components.

### Search strategies

A systematic search was performed for studies published in the period January 1990 to August 2014. Articles were retrieved through the databases, PubMed (Medline), PsycINFO (Ovid), EMBASE (Ovid), WEB of Science (ISI), and through the reference sections of published studies and relevant reviews [16]. Specific search methods were used for each database; e.g., medical subject headings (MeSH) [37] were used for the database MEDLINE (PubMED). Search details for MEDLINE (PubMED) were as follows: (((“Alcohols”[Mesh] OR “Alcohol Drinking”[Mesh]) AND “Alcohol Drinking/prevention and control”[Mesh]) AND “Adolescent”[Mesh]) AND (((“Early Intervention (Education)”[Mesh] OR “Intervention Studies”[Mesh]) OR “Evaluation Studies as Topic”[Mesh]) OR “Program Evaluation”[Mesh]) AND Randomized Controlled Trial. A similar search was conducted in WEB of Science (ISI).

In EMBASE (Ovid) and PsycINFO (Ovid), search phrases included: (School Based Intervention or Intervention or Treatment Outcomes or Primary Mental Health Prevention or Treatment Effectiveness Evaluation or Early Intervention, and (Alcohols or Binge Drinking or Alcohol Drinking Patterns or Alcohol Abuse), and (Adolescent Psychology or Adolescent Development or Adolescent Attitudes or adolescents), and (Drug Abuse Prevention or Prevention). The search was limited to *human* and *English language*.

Overall 370 published articles were identified (PubMed 75 studies, EMBASE 66 studies, WEB of Science 135 studies, and PsycINFO 94 studies) in addition to 19 studies from previously conducted meta-analyses and reviews.

**Figure 1:**
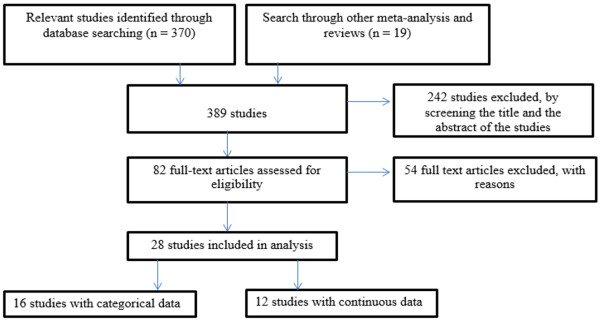
**Flow diagram for studies included in the meta-analysis.**

The process for determining the eligibility of studies to be included was conducted by two of the authors and consisted of a three-step process: 1) the title of the article was examined; 2) the abstract was reviewed; and 3) the full text was read. A total of 242 studies were excluded after screening the title and abstract of the papers. Additionally, 54 studies were eliminated after reading the full text because they did not fulfill the inclusion criteria. In addition, 20 duplicates were deleted. Thus, the final pool of included studies in the present meta-analysis consisted of 28 studies (Figure 1).

### Coding of variables

According to the project protocol, the following variables were coded for each study: descriptive information (e.g., year of publication, country), sample information (baseline characteristics like sample size, gender, and age), school level (e.g., elementary school, junior high school, or high school), program intensity (low intensity of less than 1 to 5 hours, medium intensity of 6 to 10 hours, and high intensity of 11 to 15 hours or more), and measurement characteristics like time points of follow-ups (< 3 months, 4 to 12 months, and > 13 months). Alcohol use outcomes were coded as weekly drinking (7 days’ alcohol use), monthly drinking (30 days’ alcohol use), and lifetime alcohol use (e.g., Ever used alcohol). The categorical outcomes measured the percentages of students who consumed alcohol within a defined period of time. The continuous outcomes were reported as means and standard deviations and measured the frequency of alcohol use (the number of times alcohol was consumed within a defined period of time) and the quantity of alcohol consumption (the mean number of drinks within a defined period of time).

The studies were coded by the first and the second author. To assess inter-rater reliability, 6 of the 28 studies (21%) were randomly selected and coded by two of the other authors. The main variables included in the meta-analysis calculations and moderator analyses were selected for reliability check. Inter-rater agreement was estimated as Intra-class correlation coefficients (ICC; absolute agreement). There was 100% agreement between the coders for descriptive data (school-based studies and country). ICC was 0.92 for age, 0.99 for gender (proportion of boys), and 0.99 for effect size data.

### Statistical analyses

The meta-analysis was conducted using the Comprehensive Meta-Analysis program version 2.2.057 [38]. Descriptive data were analyzed using the Statistical Package for Social Sciences (SPSS 21.0).

Because we assumed that the true effect could vary from study to study, and that factors other than sampling error could contribute to the observed variation in effect sizes (e.g., study design, sample characteristics, and type of intervention), a random effects model was used for the meta-analysis calculations. Study weights are more equal under the random effects model compared to the fixed effect model. Mean effect sizes and other meta-analysis calculations were weighted according to the inverse variance statistics comprised of both random variation and variation between studies [39].

The heterogeneity test, *Q*, was used to examine variation between studies. A significant *Q* rejects the null hypothesis of homogeneity and indicates that the variability between the effect sizes is greater than subject-level sampling alone and that moderators should be examined [40]. The ratio of true heterogeneity to the total variance across the observed effect estimates was reported as a *I*^2^ statistic. The *I*^2^ statistic ranges from 0-100% and is not affected by the number of included studies in a meta-analysis [39]. A *I*^2^ statistic close to zero indicates non-significant variance.

Two different effect-size statistics were used in the meta-analysis, standardized mean differences (Hedges’ *g*) for continuous outcomes and odds ratios (*OR*) categorical outcomes respectively.

Hedges’ *g* was calculated as the difference between the mean post-test scores of the control group and the intervention group divided by the pooled standard deviation. A positive effect size was indicated by less frequent alcohol use and less quantity of alcohol consumed in the intervention group. According to Cohen’s criteria [41], effect sizes are denoted as follows: *g* = 0.2 denotes a small effect, *g* = 0.5 a medium effect, and *g* = 0.8 a large effect [42].

Effect sizes for studies reporting categorical types of data were calculated as *OR*, which is a measure of the association between exposure and outcome [40]. A positive effect size was indicated by *OR* < 1 (fewer reporting alcohol consumption in the intervention group compared with the control group). Whereas an *OR* of 0.59 denotes a weak effect, an *OR* of 0.29 denotes a moderate effect, and an *OR* of 0.15 denotes a strong effect [43]. A value of 1.00 indicates no difference in the rate of alcohol consumption between the intervention group and the control group. A negative effect (higher consumption in the intervention group compared with the control group) was indicated by *OR* > 1.

All of the studies included examined the effectiveness of school-based programs on preventing alcohol use among adolescents. Included studies used either schools (cluster RCT) or students (RCT) as the unit for randomization. Mixed effect analyses were conducted to examine whether there was any difference between studies using RCT and cluster RCT.

To calculate the overall mean effect size, the mean of all outcomes and time points was calculated within each study before the overall mean was calculated.

When studies reported the effect of several components, like a parent component or combined components, we used the outcomes of the student intervention only [44]. When included studies used more than one intervention, we used the study as the unit for analysis and combined the effect sizes of the subgroups within each study [45]. When studies distinguished between groups by reported alcohol use at baseline, we calculated the mean alcohol use for all subgroups in both the intervention and control groups [46]. To test whether or not the observed overall effect was robust the Fail-safe *N* was calculated using Rosenthal’s procedure [47]. The fail-safe *N* is the number of studies with null-findings required to reduce a significant mean effect into a non-significant result.

Moderator analyses were only conducted when the category included at least three studies. Categorical variables (school level and program intensity) were examined by using a mixed-effects analysis and continuous moderators (age and proportion of boys) by conducting meta-regression analyses.

## Results

### Description of included studies

We identified 28 randomized studies from nine different countries, of which the majority came from the USA (61%) followed by Australia (14%). The mean publication year was 2003 (*SD* = 6.77). Demographic characteristics were only reported for the baseline samples. The total number of baseline participants was 39,289 with a mean age of 13.16 (*SD* = 1.96) years. The gender distribution was equal (50%). Sample sizes varied, ranging from 104 to 7,079 (*M* = 2017; *SD* = 1810). The majority of included studies were conducted in junior high schools (68%). Sixteen studies reported categorical measures on alcohol use (Table 1), and twelve studies reported continuous measures on alcohol use (Table 2).

**Table 1:** **Study characteristics for studies reporting categorical measures on alcohol use**

***Study***	***N***	***Gender boys***	***Age***	***School level***	***Program***	***Program intensity***	***Outcome***	***Time points in months***	***OR***_***T1***_	***OR***_***T2***_	***OR***_***T3***_	***OR***_***T4***_
Bodin et al. 2011 [60]	1752	49%	14.50	HS	ÖPP	Medium	a	12, 30	0.83	0.90		
Bond et al. 2004 [56]	2678	47%	14.00	HS	GP	High	c	12, 24, 36	0.82*	0.88	0.84*	
Caria et al. 2011 [68]	5541	51%	13.00	JHS	EU-Dap	High	a	18	0.93			
Clayton et al. 1991 [70]	1927	51%	11.50	JHS	Project DARE	High	c	6, 12, 24	1.06	1.12	1.00	
Ellickson et al. 1990 [46]	3852	49%	13.00	JHS	Project ALERT	High	a, b	3, 12, 15	0.99	1.03	0.99	
Furr-Holden et al. 2004 [49]	566	54%	13.00	JHS	GBG	High	c	24	1.04			
Griffin et al. 2009 [52]	178	54%	13.50	JHS	The Brave	High	b	12	0.13***			
Koning et al. 2009 [44]	2570	51%	12.70	JHS	HSD	Medium	a	8, 12	0.96	0.80*		
McBride et al. 2004 [71]	2343	-	13.00	JHS	SHAHRP	High	a, b	8, 12, 18	0.80	0.80*	0.87	
McCambridge et al. 2011 [50]	416	55%	17.50	HS	MI	Low	C	3, 12	1.22	1.04		
Ringwalt et al. 1991[54]	1270	48%	10.40	JHS	Project DARE	High	C	3	1.22			
Ringwalt et al. 2009 [59]	6028	49%	10.50	JHS	Project ALERT	High	b, c	24	1.08			
Schinke et al. 2000 [67]	1396	51%	10.28	ES	LST	High	A	6, 18, 30, 42	0.66***	0.78	0.80	0.68**
Spoth et al. 2002 [55]	919	52%	10.50	JHS	LST	High	C	12	0.94			
St. Pierre et al. 2005 [72]	1649	50%	10.50	JHS	Project ALERT	Medium	B	24	1.09			
Sun et al. 2008 [69]	2064	53%	15.70	HS	TND-4	Medium	B	12	1.00			

Note. a = Report changes in weekly alcohol use, b = Report changes in monthly alcohol use, c = Report changes in lifetime alcohol use. OR = Odds Ratio. JHS = Junior High School; HS = High School; ES = Elementary School. EU-Dap = European Drug Abuse Prevention; GBG = Good Behavior Game; GP = Gatehouse Project; HSD = Healthy School and Drugs; LST = Life Skills Training; MI = Motivational Interview; ALERT = Adolescent Learning Experiences in Resistance Training; DARE = Drug Abuse Resistance Education; ÖPP = Örebro Prevention Programme; The BRAVE = Building Resiliency and Vocational Excellence; TND-4 = Project Towards No Drugs Abuse. *p < .05. **p < .01 ***p < .001.

**Table 2:** **Study characteristics for studies reporting continuous measures on alcohol use**

***Study***	***N***	***Gender boys***	***Age***	***School level***	***Program***	***Program intensity***	***Outcome***	***Time points in months***	***Hedges’g***_***T1***_	***Hedges’g***_***T2***_	***Hedges’g***_***T3***_
Caplan et al. 1992 [66]	282	55%	12.00	JHS	PDYP	High	c	3	0.33*		
Clark et al. 2010 [62]	2467	49%	16.72	HS	Project SUCCESS	Medium	b	1, 12	0.01	0.04	
D’Amico et al. 2002 [45]	300	42%	16.00	HS	DARE & RSTP	Low	a	2, 6	0.06	0.27***	
Newton et al. 2009 [57]	764	60%	13.08	JHS	CLIMATE^2^	High	a	1, 6	0.12	0.36***	
Peleg et al. 2001 [64]	1000	44%	15.50	JHS	LST	Medium	c	12, 24	1.17***	0.95***	
Reddy et al. 2002 [53]	4776	51%	11.90	JHS	HRIDAY	High	c	12	0.18***		
Shope et al. 1992 [63]	2589	-	10.50	ES	AMPS	Medium	c	6, 18, 30	0.06	0.12	0.11
Vogl et al. 2009 [61]	1466	59%	13.00	JHS	CLIMATE^1^	Medium	a	1, 6, 12	0.01**	0.02	0.04
Warren et al. 2006 [48]	4734	53%	12.50	JHS	keepin’it R.E.A.L	Medium	b	18	0.07*		
Werch et al. 2005 [58]	604	44%	15.24	HS	Project SPORT	Low	c	3, 12	0.22**	0.10	
Werch et al. 1996 [51]	104	44%	13.80	JHS	STARS	Medium	b	1, 2	0.21	0.46*	
Wilhelmsen et al. 1994 [65]	915	-	13.50	JHS	Young and alcohol	Medium	c	3	0.04		

Note. a = Report changes in weekly alcohol use, b = Report changes in monthly alcohol use, c = Report changes in lifetime alcohol use. JHS = Junior High School; HS = High School; ES = Elementary School. AMPS = Alcohol Misuse Prevention Study; CLIMATE = ^1^Alcohol Course, ^2^Alcohol and Cannabis course; DARE = Drug Abuse Resistance Education; HRIDAY = Health Related Information and Dissemination Among Youth (Hindu word for “Heart”); R.E.A.L = Refuse, Explain, Avoid, Leave; LST = Life Skills Training; PDYP = Positive Youth Development Program; RSTP = Risk Skills Training Program; SUCCESS = Schools Using Coordinated Community Efforts to Strengthen Students; STARS = Start Taking Alcohol Risks Seriously. *p < .05. **p < .01 ***p < .001.

The majority of the included studies used prevention strategies addressing normative and social influences. In addition, some interventions provided alcohol education and life skills training, including coping strategies and problem solving skills [44, 45, 48–51]. Furthermore, most of the studies measured outcomes like cigarette/marijuana and drug use, in addition to alcohol use [45, 48, 49, 52–55]. Two studies also assessed bullying and harmful behavior [50, 56].

### Quality of studies

All included studies used a randomized control design. Two of the 16 studies using categorical measures, used the students as the unit for randomization (RCT) while 14 used the schools as the unit of assignment (cluster RCT). Among the 12 studies reporting the continuous outcomes, four studies used students and eight used the schools as the unit of assignment. Mixed effect analyses comparing the two groups (RCT versus cluster RCT) showed no significant differences for studies reporting the categorical outcomes (*Q* = 0.79, df = 1, *p* = .37) or continuous outcomes (*Q* = 1.56, df = 1, *p* = .21). The methods used for randomization included use of computer or online systems [50, 51, 57–59], coin tossing [60], simple random sampling (e.g., random assignment by an independent researcher) [44, 56, 61], and random assignment of numbers to the students to further assigned them to the condition [52]. In the remaining 13 studies the method of randomization was unclear [45, 46, 48, 49, 53–55, 62–72]. One study additionally calculated and assumed random allocation of schools [71]. Students were blind to group assignment in two studies [59, 61].

Follow-up assessments were conducted within a time range from one to 42 months, distributed among 12 different follow-up periods. Most common was one year follow-up (*K* = 15) followed by two year (*K* = 6), 18 months (*K* = 6), six month (*K* = 6), and three month follow-up periods (*K* = 6). Attrition rates were reported by seven of the 12 studies reporting continuous outcomes, and by ten of the 16 studies reporting categorical outcomes. Attrition rates varied from 5% to 52%.

### Intervention effects

For studies reporting continuous outcomes, the overall meta-analysis calculations resulted in a small and significant effect in favor of the intervention ( = 0.22, z = 2.99, *p* < .01) (Table 3). The value of the file drawer statistic indicated that at least 301 unpublished studies would be needed to reduce the obtained effect to a non-significant finding, which is considerably higher than the suggested limit (5 K + 10 = 70). The overall effectiveness for frequency of alcohol use was small, and not significant ( = 0.09, z = 1.94, *p* = .053). The intervention effects for the quantity of alcohol consumed was small and significant in favor of the interventions ( = 0.29, z = 2.46, *p* < .01). The overall mean effect size for studies reporting categorical outcomes was not significant ( = 0.94, z = −1.15, *p* = .25). The tests of heterogeneity showed a significant variance between the included studies, indicating that moderators may be present.

**Table 3:** **Overall effect sizes and combined outcomes by different time points presented for studies reporting continuous and categorical measures**

	***Studies reporting continuous measures***							***Studies reporting categorical measures***						
	***K***	***N***		95% CI	***Q***	***df***	***I*** ^***2***^	***K***	***N***		95% CI	***Q***	***df***	***I*** ^***2***^
Overall effect size	12	16279	0.22**	0.08-0.36	184.11***	11	94.03%	16	23010	0.94	0.85-1.04	38.08***	15	60.61%
Alcohol use:														
<3 months	8	6617	0.10**	0.03-0.17	10.66	7	34.35%	3	5763	1.18*	1.00-1.40	0.82	2	0.00%
4-12 months	8	10479	0.27*	0.03-0.52	239.19***	7	97.07%	11	16409	0.86*	0.75-0.99	29.57***	10	66.18%
>13 months	3	6617	0.37	−0.14-0.88	113.88***	2	98.24%	10	18177	0.95	0.89-1.02	9.525	9	5.52%

Note. Random effect model. *k* = number of studies; *N* = total number of participants;  = mean Hedges’g;  = mean Odds Ratio; *Q* = test of heterogeneity; 95% CI = confidence interval; df = degrees of freedom; *I* ^*2*^ = proportion of observed dispersion. *p < .05 **p < .01 ***p < .001.

#### Primary outcomes

Different analyses were conducted to estimate the effect of preventive alcohol interventions over time (Table 3) and to compare the effect of the three primary outcomes that included weekly alcohol use, monthly alcohol use, and lifetime alcohol use for studies reporting the categorical outcomes (Table 4) and for the studies reporting continuous outcomes (Table 5).

**Table 4:** **Intervention effects on adolescent alcohol use of combined time points for studies reporting categorical measures**

	***Studies reporting categorical measures***						
	***k***	N		95% CI	***Q***	***df***	***I*** ^***2***^
Weekly drinking	6	10140	0.86***	0.78-0.95	3.71	5	0.00%
Monthly drinking	6	11544	0.92	0.75-1.12	22.05***	5	77.33%
Lifetime drinking	7	11725	1.04	0.93-1.17	11.02	6	45.53%

Note. Random effect model. *k* = number of studies; N = total number of participants;  = mean Odds Ratio; 95% CI = 95% confidence interval; *Q* = test of heterogeneity; df = degrees of freedom; *I* ^*2*^ = proportion of observed dispersion. *p < .05 **p < .01 ***p < .001.

**Table 5:** **Intervention effects for studies reporting continuous measures for frequency and quantity of alcohol use**

	***Frequency of Alcohol Use***							***Quantity of Alcohol Use***						
	***k***	N		95% CI	***Q***	***df***	***I*** ^***2***^	***k***	N		95% CI	***Q***	***df***	***I*** ^***2***^
Weekly drinking	0	-	-	-	-	-	-	3	3570	0.13*	0.01-0.25	3.98	2	49.70%
Monthly drinking	2	2119	0.07	−0.05-0.20	1.76	1	43.18%	2	4838	0.13	−0.09-0.35	1.81	1	44.69%
Lifetime drinking	2	3536	0.10	−0.06-0.27	4.25	1	76.45%	3	2216	0.50	−0.18-1.17	88.75***	2	97.75%

Note. Random effect model. *k* = number of studies; N = total number of participants;  = mean Hedges’g; 95% CI = 95% confidence interval; *Q* = test of heterogeneity; df = degrees of freedom; *I* ^*2*^ = proportion of observed dispersion. *p < .05 **p < .01 ***p < .001.

#### Intervention effects < 3 months

Within the measure of a short-time interval (< 3 months), studies reporting continuous measures showed a small but significant positive effect size of alcohol preventive interventions. Studies reporting categorical outcomes showed a small but negative effect size on alcohol use, indicating that the intervention groups scored higher on alcohol use as compared to the control group (see Table 3). The test of heterogeneity was not significant, but this could be due to low power as there was a small number of included studies.

#### Intervention effects between 4–12 months

The effect sizes for the follow-up period from four to 12 months were small and significant for both OR and Hedges’ g, favoring the preventive intervention programs. Both heterogeneity tests were significant (see Table 3).

#### Intervention effects > 13 months

Long-term follow-up (> 13 months) showed non-significant effect sizes for the interventions. The level of heterogeneity was significant in studies reporting continuous outcomes but not significant among studies reporting categorical outcomes (see Table 3).

#### Weekly alcohol use

Overall nine studies measured weekly alcohol use [44–46, 57, 60, 61, 67, 68, 71]. The overall effect sizes were small and significant, demonstrating a positive intervention effect. The heterogeneity test was not significant (see Table 4 and Table 5).

#### Monthly alcohol use

Ten studies measured monthly alcohol use [46, 48, 51, 52, 58, 59, 62, 69, 71, 72]. The overall effect sizes were not significant. The test of heterogeneity within studies reporting continuous changes in monthly alcohol use was not found to be statistically significant, however, it was significant within studies reporting categorical outcomes (see Table 4).

### Lifetime alcohol use

Twelve studies measured the lifetime use of alcohol [49, 50, 53–56, 59, 63–66, 70]. The overall effect sizes were not significant for *OR* or Hedges’ *g.* The level of heterogeneity was significant between studies reporting the alcohol quantity, but not significant in studies reporting the frequency of alcohol use (Table 5) or among studies reporting the categorical outcomes (Table 4).

### Moderator analysis

The moderator analysis comparing different school levels did not show significant differences between interventions implemented at junior high school or high school (Table 6). Because there were only two studies conducted at elementary schools, these were not included in this analysis [63, 67].

**Table 6:** **Moderator analysis for school level and program intensity for studies reporting continuous and categorical measures**

	***Studies reporting continuous measures***							***Studies reporting categorical measures***						
	***k***		***95% CI***	***Q***	***df***	***I*** ^***2***^	***Total between Q***	***k***		***95% CI***	***Q***	***df***	***I*** ^***2***^	***Total between Q***
School level:							0.80							0.00
Junior high school	7	0.12***	0.05-0.19	14.41*	6	58.42%		8	0.91	0.77-1.07	25.24***	7	72.26%	
High school	4	0.35	− 0.15-0.85	143.91***	3	92.92%		4	0.91	0.80-1.03	2.93	3	0.00%	
Program intensity:							0.07							0.09
Medium (6 to 10 hours)	7	0.23	− 0.00-0.46	180.11***	6	96.67%		3	0.90	0.76-1.07	0.47	2	0.00%	
High (11 to >15 hours)	3	0.20***	0.13-0.26	1.34	2	0.00%		12	0.93	0.82-1.06	36.16***	11	69.58%	

Note. Mixed effect analysis. *k* = number of studies;  = mean Hedges’g;  = mean Odds Ratio; 95% CI = 95% confidence interval; *Q* = test of heterogeneity; df = degrees of freedom; *I* ^*2*^ = proportion of observed dispersion. *p < .05 **p < .01 ***p < .001.

The moderator analysis between different levels of program intensity showed no significant differences between medium intensity (6 to 10 hours) or high intensity programs (11 to >15 hours) (Table 6). Low intensity programs were not included in the moderator analysis as there was only one study reporting categorical outcomes [50] and only two studies reporting continuous outcomes [45, 58].

#### Meta regression

Meta regressions were conducted to examine the influence of the moderator variables, age and gender, on the effectiveness of preventive alcohol interventions.

Gender was coded as the proportion of boys in the study samples. The meta-regression results were not significant for gender in studies reporting continuous outcomes (*β*_*1*_ *= −* 0.02, *z = −*1.23, *p =* .22), nor in studies reporting categorical outcomes (*β*_*1*_ *= −*0.01, *z* = − 0.45, *p = .*65).

Similarly, age was not found to be a significant moderator, both for studies reporting continuous outcomes (*β*_*1*_ *=* 0.04, *z* = − 0.98, *p =* .33) and for studies reporting categorical outcomes (*β*_*1*_ *= −* 0.01, *z* = − 0.45, *p =* .65).

## Discussion

The aim of the current meta-analysis was to estimate the effectiveness of school-based preventive programs on alcohol use among adolescents. To our knowledge, this is the first meta-analysis on this topic that exclusively included studies with randomized designs. Furthermore, the aim was to assess the effectiveness of the interventions over time and to examine whether the effect of the intervention differed according to the different school levels or level of program intensity.

The overall effect size among studies reporting continuous outcomes was small but significant, indicating that alcohol prevention interventions may have a positive influence on alcohol use among adolescents. However, the overall effect size of studies reporting categorical outcomes was weak and not significant. Categorization of continuous variables is common in health sciences and medical research, but there is a cost to dichotomizing continuous variables [73]. Studies that report categorical or dichotomous data lose one-third to two-thirds of the information on the variance of the sample [74]. This reduces the calculated effect sizes and, thus, the effectiveness of the intervention may be underestimated when using this approach. This might explain why there was no significant overall effect among studies reporting the categorical outcomes. Furthermore, this analysis showed a small but significant effect on adolescents’ weekly alcohol use. The effectiveness on monthly alcohol use was small and in a desired direction favoring the preventive programs in studies reporting the continuous outcomes, whereas this effect was not significant among studies reporting categorical data. The prevention programs did not affect general alcohol use among adolescents, measured by lifetime alcohol use, a finding that was expected. Outcomes measuring adolescents’ lifetime alcohol use include whole samples, of which the majority has not started to drink alcohol yet.

Results measuring the effectiveness of the preventive interventions after a short term follow-up (< 3 months) were mixed. The generalized preventive effect for studies reporting continuous outcomes was positive and in favor of the preventive program. This result is in line with other studies that have found that school-based alcohol interventions can be an effective approach to preventing alcohol use in the short term [6, 12]. Furthermore, the heterogeneity test was not significant, suggesting no significant variance between those studies. However, among studies reporting categorical measures, the results indicated a higher alcohol use rate in the intervention group as compared to the control group, which may indicate an adverse effect of the interventions. This finding should nonetheless be interpreted with caution, since only three of the included studies reported categorical outcomes at 3 months. Aside from this finding, all effects were in favor of the interventions although the effects were small.

The overall impression of the results was that the prevention effects on alcohol use are significant and positive, in addition to increasing over time for the follow-up period four to twelve months. The effect of school-based prevention was generally positive on adolescents’ alcohol use (weekly and monthly), however, such positive effect was not measured for lifetime drinking. This could indicate that preventive programs fail to postpone the onset of alcohol use or that the number of adolescents drinking alcohol in either group may be too low to demonstrate a statistically significant difference between the two groups. An implication of this finding is that studies should follow the adolescents for longer periods of time, at least long enough for experimentation of alcohol use to occur. This result held for studies reporting both continuous and categorical outcome measures.

Research has demonstrated that brief program duration of less than four months is generally as effective as those with a longer duration [21]. Additionally, a recently conducted meta-analysis concluded that brief school-based alcohol interventions (shorter than five hours of duration) may be effective in reducing adolescents alcohol use [20]. On the other hand, research has also showed that prevention programs seem to be more successful when they are maintained over several years, interactive [17], and incorporate more than one strategy; e.g., addressing social norms, building social resistance skills, providing booster-sessions, and using peer-leaders [29]. Unfortunately, there was a general lack of detailed information on intervention strategies used among the included studies. Evaluation studies should provide more detailed information about potential moderators like implementation process, program fidelity, and attrition rate that will provide valuable information. This issue has also been raised elsewhere [16, 75].

The long-term results from this meta-analysis show no significant differences between intervention and control groups beyond the one year follow-up. The discontinuity in the development of drinking behavior during adolescence might explain the challenges that preventive intervention faces in reaching long-term effects [76]. Some evidence from school-based prevention research indicates that intervention programs do not reduce alcohol use in the long term (> 12 months) [77]. However, a review of the long-term effectiveness of alcohol prevention programs provides evidence of reduced alcohol use for up to 15 years after program implementation [78].

The majority of included studies was implemented at junior high school level. The moderator analysis in this meta-analysis showed no significant effect between different school levels. Furthermore, the moderator analysis did not show any statistically significant differences in the comparison of low, medium, and high intensity programs. Both findings are in line with the previous work conducted by Tobler and colleagues [17]. They eliminated grade as an effective program predictor based on non-significant findings in addition to report no significant difference between high and low intensity of programs [17]. As such, it is promising that treatment efforts with medium intensity do seem to obtain treatment effects comparable to programs of higher intensity due to possible cost-benefit gains. A national survey conducted among US schools showed that the effectiveness of preventive practices would be improved if schools increased the intensity of program activity [79].

Studies suggest that primary prevention programs for alcohol use should occur prior to sixth grade, particularly for the group at high risk of early use [80]. Unfortunately, there were only two included studies in our analysis that reported on elementary schools and, therefore, we were not able to confirm this finding.

### Implications

Our findings show that the preventive effects of school-based preventive interventions on adolescent alcohol use are small but generally positive, regardless of the intensity of the program. It is important to bear in mind that even small effects can make a difference. School-based alcohol interventions are found to be cost effective because they may avert costs associated with harmful drinking. Research by Caulkins and colleagues [9] estimated that even small effect sizes in universal prevention interventions could lead to important savings for the society. Implementing universal preventive interventions within schools, where a large number of adolescents are reached, can lead to positive health outcomes within the society as further suggested by this study. Delaying alcohol debut among adolescents is important and has several possible health gains such as well-being and social development, important to both the public and the individual [10].

This study could not find any evidence to suggest which school level is preferable for implementing a preventive intervention or which level of program intensity would be most efficacious. Neither were age nor gender found to be moderators for effectiveness, however, the overall effectiveness of school-based preventive alcohol interventions for adolescents was measured as preferable and significant up to a year from implementation. After one year, our findings show no significant results. Only three studies with continuous measures reported long-term treatment effect, whereas 10 studies reported no treatment effect on categorical measures of alcohol use.

### Limitations

This study has some limitations that should be taken into consideration when interpreting the results. The literature search resulted in relatively few studies that fulfilled the inclusion criteria. There were considerable differences in sample sizes between the studies, although the total number of adolescents included in the analysis is fairly large. Additionally, there was a significant heterogeneity between the studies, while the moderator variables could not explain this variability. This indicates widely dispersed results, meaning that the true effects most likely do vary [39]. In addition, the moderator analyses included only a small number of studies, which led to low statistical power, and the variance in age and gender between studies was small. A non-significant *p*-value should not be taken as evidence that the effect sizes are consistent, since the lack of significance may be due to low power [39]. One strong aspect of this meta-analysis is that we only included randomized controlled studies. This provided stronger evidence of the interventions’ effectiveness, since randomized studies have the highest possible internal validity.

## Conclusion

Our findings show that school-based interventions overall have a small but positive effect on alcohol use among adolescents up to one year after program implementation for both boys and girls independent of age. Small effect sizes can make a difference, especially when it comes to universal preventive interventions. Alcohol education should be considered as part of a wider policy approach and should be based on educational practices that have been proven to be effective [81]. Interventions should be focused on specific ingredients that lead to preventing alcohol use among adolescents. Future research needs to continue developing and testing the implementation of interventions already demonstrated to reduce alcohol use among adolescents. The evidence base related to school-based alcohol interventions must continue to develop in order to improve their effectiveness.
